# Long non-coding RNA CHCHD4P4 promotes epithelial-mesenchymal transition and inhibits cell proliferation in calcium oxalate-induced kidney damage

**DOI:** 10.1590/1414-431X20176536

**Published:** 2017-11-13

**Authors:** C. Zhang, J. Yuan, H. Hu, W. Chen, M. Liu, J. Zhang, S. Sun, Z. Guo

**Affiliations:** 1 Department of Nephrology, Changhai Hospital, Second Military Medical University, Shanghai, China; 2 Department of Medical Genetics, Second Military Medical University, Shanghai, China; 3 Department of Urology, Changhai Hospital, Second Military Medical University, Shanghai, China

**Keywords:** Kidney calculi, Long non-coding RNAs, Fibrosis, Cell proliferation

## Abstract

Kidney stone disease is a major cause of chronic renal insufficiency. The role of long non-coding RNAs (lncRNAs) in calcium oxalate-induced kidney damage is unclear. Therefore, we aimed to explore the roles of lncRNAs in glyoxylate-exposed and healthy mouse kidneys using microarray technology and bioinformatics analyses. A total 376 mouse lncRNAs were differentially expressed between the two groups. Using BLAST, 15 lncRNA homologs, including AU015836 and CHCHD4P4, were identified in mice and humans. The AU015836 expression in mice exposed to glyoxylate and the CHCHD4P4 expression in human proximal tubular epithelial (HK-2) cells exposed to calcium oxalate monohydrate were analyzed, and both lncRNAs were found to be upregulated in response to calcium oxalate. To further evaluate the effects of CHCHD4P4 on the cell behavior, we constructed stable CHCHD4P4-overexpressing and CHCHD4P4-knockdown HK-2 cells. The results showed that CHCHD4P4 inhibited cell proliferation and promoted the epithelial-mesenchymal transition in kidney damage and fibrosis caused by calcium oxalate crystallization and deposition. The silencing of CHCHD4P4 reduced the kidney damage and fibrosis and may thus be a potential molecular target for the treatment of kidney stones.

## Introduction

The formation of kidney stones is a common urological disorder, and the number of patients presenting with this disorder is increasing. Calcium oxalate (CaOx) is a major constituent, accounting for more than 80% of kidney stones (1). In addition, acute oxalosis (such as in glyoxylate poisoning) can induce acute kidney damage due to the renal tubular blockage caused by the deposition of the CaOx crystals (2).

Kidney stones are associated with a significant loss of kidney function and are frequently accompanied by interstitial fibrosis, which is a common result of various kidney diseases (3,4). In our previous study, we found that the epithelial-mesenchymal transition (EMT) occurred in a mouse model injected with glyoxylate (5). Several other studies have also suggested that kidney fibrosis was caused by the transition of renal epithelial cells to mesenchymal cells, which is a phenomenon related to cell proliferation. For example, cultured mouse renal tubular epithelial cells that were stimulated by transforming growth factor-β1 (TGF-β1) were inhibited in the G2/M cell cycle, and the production of profibrotic cytokines that can stimulate the proliferation and transition of pericytes to myofibroblasts was induced (6). In mouse models of experimentally induced renal fibrosis, the functional consequence of the EMT during fibrotic injury is arrested in the G2 phase of the cell cycle (7).

Long non-coding RNAs (lncRNAs) are a subgroup of non-coding RNAs that are greater than 200 nucleotides in length. In recent years, multiple studies have indicated that lncRNAs play key roles in a variety of biological processes, such as proliferation and apoptosis, through complex mechanisms (8). Several reports have highlighted the role of lncRNAs in the pathogenesis of the EMT. For example, Zhou et al. found that the lncRNAs np_5318/np_17856 were associated with the TGF-β/Smad3-mediated renal inflammation and fibrosis in a Smad3 knockout mouse model of unilateral ureteral obstructive nephropathy (9). Additionally, they proved that the treatment of an obstructed kidney with lncRNA-Arid2-IR shRNA blunted the NF-κB-driven renal inflammation without any effect on the TGF-β/Smad3-mediated renal fibrosis (10). Our previous studies have shown that EMT appears in tubular epithelial cells during the early period of kidney stone formation in a crystalline kidney injury, which initiates the process of renal fibrosis (5). Long non-coding RNAs can play a regulatory role in the development of EMT, but it is unknown whether lncRNAs are involved with or influence EMT in tubular epithelial cells when induced by crystallization; we hypothesized that EMT might be influenced by lncRNAs in human renal proximal tubular epithelial cells (HK-2) *in vitro*. Furthermore, we explored the underlying mechanisms, which may provide new insights into the pathogenesis and treatment of tubular EMT and renal tubular interstitial fibrosis caused by kidney stones.

## Material and Methods

### Experimental models

We established an experimental mouse model of calcium oxalate-induced kidney damage by intraperitoneally injecting C57BL/6J mice (age: 8 weeks, weight: 26–30 g) with glyoxylate (100 mg/kg) for 5 days as described previously (5). The control animals were intraperitoneally injected with 0.9% NaCl for 5 days. All procedures were approved by the Institutional Animal Care and Use Committee of the Second Military Medical University. As an *in vitro* model, HK-2 cells were exposed to calcium oxalate monohydrate (COM; 200 µg/mL) for 24 h. Glyoxylate sodium salt was purchased from Tokyo Chemical Industry (Japan). Calcium oxalate monohydrate was purchased from Sigma (USA). HK-2 cells were donated by Professor Xiong Jun, from the Anatomy Laboratory, The Second Military Medical University (China).

### Microarray hybridization and data analysis

Kidney samples from 3 experimental mice and 3 control mice were extracted and used to synthesize double-stranded complementary DNA, which was labeled and hybridized to 8×60 K lncRNA Agilent Genomic Expression Arrays. The gene chips were washed, stained, and then scanned with an Axon GenePix 4000B microarray scanner (Molecular Devices, USA). The raw data were extracted as paired files using the NimbleScan software (version 2.5; Roche NimbleGen, USA). The hierarchical clustering of the differentially expressed lncRNAs was performed using the Cluster 3.0 and Java Treeview (USA). The Gene Ontology (GO) annotations for the microarray genes were downloaded from the NCBI and Gene Ontology databases. A pathway analysis was carried out using the KEGG database.

### Von Kossa staining

The von Kossa method for quantifying calcium crystal formation and deposition was performed as described previously (5).

### Cell fractionation assay

Cytoplasmic and nuclear RNA were acquired using the Cytoplasmic and Nuclear RNA Purification Kit (Norgen, Canada) according to the manufacturer's instructions. Briefly, 1×10^7^ HK-2 cells were harvested and incubated with a lysis solution for 5 min on ice. Then, the cells were centrifuged at 500 *g* for 3 min at 4^o^C, the supernatant was kept for assessing the cytoplasmic RNA, and the pellet was used for nuclear RNA extraction.

### Silencing and overexpressing lncRNA CHCHD4P4

The siRNA for CHCHD4P4 (5′-CAUGGAUUGAUACUACCAATT-3′) and a negative-control siRNA (5′-UUCUCCGAACGUGUCACGUdTdT-3′) were purchased from GenePharma (China). The *CHCHD4P4* gene was cloned into the expression vector pcDNA3.1 (GenScript, China). All plasmid vectors (pcDNA3.1-CHCHD4P4 and an empty vector for transfection) were extracted using the DNA Miniprep Kit (Axygen Scientific, Inc., USA).

### Western blotting and real-time PCR

The cell protein lysates were separated by 10% sodium dodecyl sulfate-polyacrylamide gel electrophoresis and transferred to 0.2-μm NC membranes (Bio-Rad, USA). The nonspecific binding sites were blocked overnight using 5% non-fat milk, after which the membranes were incubated with specific antibodies. The β-Actin antibody was used as a control. The anti-E-cadherin (Cat. No. ab-18203) and anti-vimentin (Cat. No. ab-1416) antibodies (1:1000) were purchased from Santa Cruz Biotechnology (USA). The total RNA was isolated from the tissues or cultured cells using TRIzol (Invitrogen, USA). For real-time PCR, the RNA was reverse-transcribed to cDNA using a Reverse Transcription Kit (Takara, Japan). Real-time PCR analyses were performed using Power SYBR Green according to the manufacturer's instructions (Takara, China). The reactions were carried out on the StepOne™ Real-Time PCR System (Applied Biosystems, USA). The sequences of the primers are listed in Table 1.

**Table 1.** Real-time RT-PCR primers.

Genes	Sequences
β-actin	
Sense	5′-TGTGTTGGCGTACAGGTCTTTG-3′
Anti-Sense	5-′GGGAAATCGTGCGTGACATTAAG-3′
CHCHD4P4	
Sense	5′-ACGAGGAGCATGGATTGATA-3′
Anti-Sense	5′-GGGATAGAGGTCTGGGTATTTC-3′
ZEB1	
Sense	5′-ACTCTGATTCTACACCGC-3′
Anti-Sense	5′-TGTCACATTGATAGGGCTT-3′
Vimentin	
Sense	5′-CCTGAACCTGAGGGAAACTAA-3′
Anti-Sense	5′-GCAGAAAGGCACTTGAAAGC-3′
Snail	
Sense	5′-TGCGTCTGCGGAACCTG-3′
Anti-Sense	5′-GGACTCTTGGTGCTTGTGGA-3′
AU015836	
Sense	5′-GCTGCAAGCTGTTAGTTGGG-3′
Anti-Sense	5′-GGGGATCATTGGGCTCGT-3′
GAPDH	
Sense	5′-GGCATCTTGGGCTACACT-3′
Anti-Sense	5′-GCCGAGTTGGGATAGGG-3′

### Cell proliferation assay

Cell proliferation was assayed using a Cell Counting Kit-8 (Dojindo, Japan) and a 5-ethynyl-2′-deoxyuridine (EDU) Immunofluorescence Kit (Ruibo, China) according to the manufacturer's instructions.

### Immunohistochemistry and immunofluorescence assays

Immunohistochemistry was performed as described previously (5). For the immunofluorescence, the cells were fixed on coverslips using ice-cold 4% paraformaldehyde for 30 min, followed by three washes with phosphate-buffered saline (PBS). The cells were then permeabilized with 0.5% Triton-PBS for 10 min, followed by treatment with 4% H_2_O_2_ in PBS for quenching the endogenous peroxidase activity. The nonspecific background was blocked using a serum-free protein block (Dako, Denmark). Then, the cells were incubated with primary antibodies against E-cadherin or vimentin (1:100) at room temperature for 2 h, followed by three washes with PBS. This was followed by incubation with the anti-mouse IgG and washing with 0.1% Triton-PBS. The cells were then stained with DAPI, followed by three washes with 0.1% Triton-PBS. Random fields were digitized using a digital camera attached to a Nikon microscope (Japan).

### Assessment of apoptosis and cell cycle progression

Apoptosis and the cell cycle progression were assessed using a Cell Cycle and Apoptosis Analysis Kit (Cat. No. C1052; Beyotime, China) and an Annexin V FITC Apoptosis Detection Kit I (Cat. No. 556547; BD Pharmingen, USA) according to the manufacturers' instructions using a flow cytometer (BD Biosciences, USA). The data were analyzed using modified software (BD Biosciences).

### Statistical analysis

Statistical analyses were performed using the SPSS software version 17.0 (SPSS, USA). Statistical significance was determined using one-way analysis of variance (ANOVA) and Student's *t*-test. The results were considered statistically significant at P<0.05. Data are reported as means±SD of three independent experiments.

## Results

### Generation of the kidney stone disease mouse model

**Figure 1.**
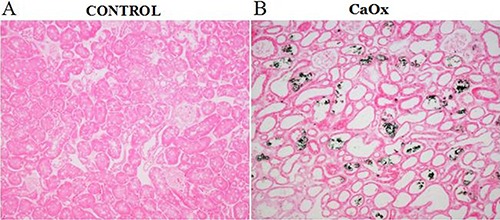
Von Kossa-stained sections of kidneys from mice in the control and experimental groups (experimental group was injected with glyoxylate for 5 days). Significantly higher calcium oxalate (CaOx) deposition was observed in the experimental group (*B*) than in the control group (*A*). Magnification: 200×.

While calcium crystal deposition was undetectable in the control group (Figure 1A), several refractive irregular crystals were observed in the junction of the renal cortex and medulla in the experimental group after 5 days of glyoxylate administration (Figure 1B). Additionally, tubular epithelial cells were deformed and focally sloughed from the basement membranes. These findings indicated the successful establishment of the model.

### lncRNA expression in the mouse kidneys was induced by glyoxylate administration

**Figure 2.**
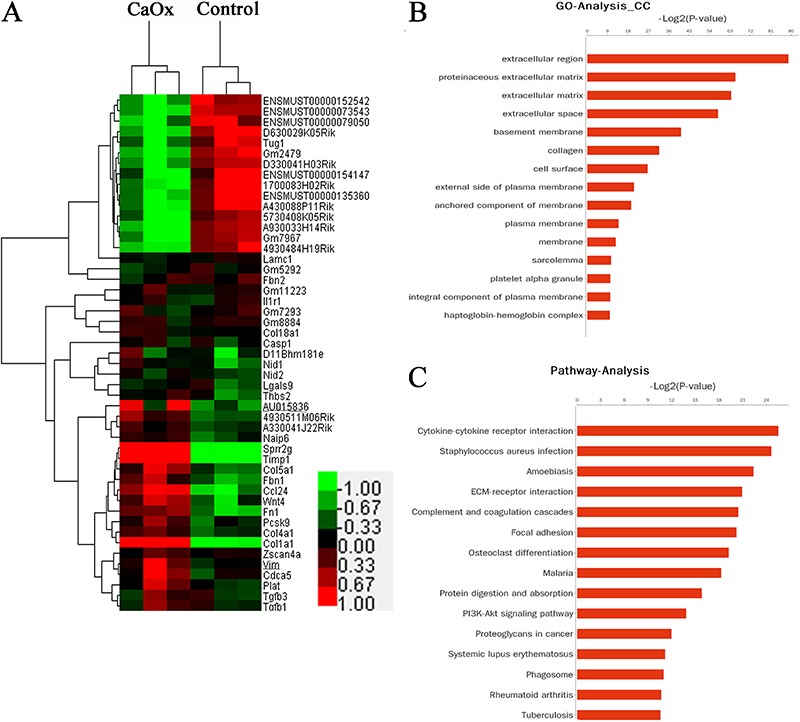
Differential expression of lncRNAs between the mouse kidneys exposed to glyoxylate and those from the control group. Hierarchical clustering analysis of 2165 mRNAs and 376 lncRNAs (*A*) that were differentially expressed between the mouse kidneys with calcium oxalate (CaOx) crystals and those from the control group (fold-change >1.5; P<0.05). Gene Ontology (GO) categories were based on the cellular components of differentially expressed genes (*B*). P<0.01 and FDR <0.05 were used as the thresholds for selecting the significant GO categories. KEGG pathway analysis of the differentially expressed genes (*C*). P<0.01 and FDR<0.05 were used as the thresholds for selecting the significant KEGG pathways; LogP is the logarithm of the P value.

The hierarchical clustering showed systematic differences in the expression of lncRNAs and protein-coding RNAs between the kidneys of the mice administered glyoxylate and those in the control group (Figure 2A). We identified 2165 differentially expressed genes; among these, 1421 genes were upregulated, and 744 genes were downregulated in the glyoxylate-exposed mice. A total of 376 lncRNAs were expressed in the CaOx-exposed group, of which 154 were upregulated, and 222 were downregulated compared to those in the control group. These genes were classified into different functional categories according to the GO terms for the cellular components. We found that these differentially expressed transcripts were mainly associated with the functions of the proteinaceous extracellular matrix (ECM), basement membrane, and collagen (Figure 2B). The main KEGG pathways for the differentially expressed genes involved cytokine-cytokine receptor interactions, ECM-receptor interactions, complement and coagulation cascades, and focal adhesion (Figure 2C). Thus, both the GO and pathway analyses suggested that the majority of the differentially expressed mRNAs may be related to the extracellular matrix.

### CHCHD4P4 was upregulated in HK-2 cells that were exposed to COM

**Figure 3.**
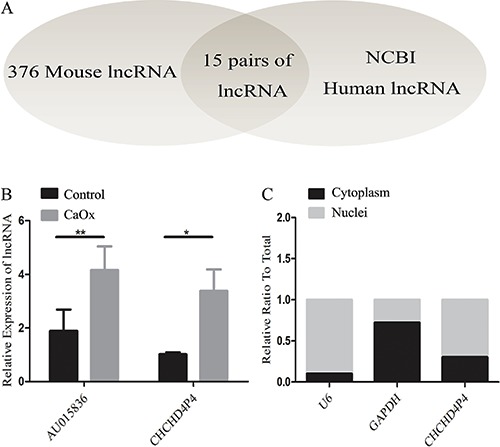
lncRNA CHCHD4P4 is upregulated in the HK-2 cells that were exposed to calcium oxalate monohydrate. *A*, Application of the BLAST algorithm in mouse and human lncRNAs identified 15 pairs of homologous lncRNAs (Table 2). *B*, Validation of the differential expression of CHCHD4P4 and AU015836 in the CaOx models and control groups using RT-PCR. *C*, Nuclear and cytoplasmic CHCHD4P4 RNAs from the HK-2 cells. Data are reported a means±SD. *P<0.05, **P<0.01 (*t*-test).

To analyze the human lncRNAs that are homologs of the mouse lncRNAs, we applied the BLAST algorithm to mouse lncRNAs (sequences longer than 200 bp retrieved from the NCBI mouse genome GRCM38.p2) and human lncRNAs (sequences longer than 200 bp retrieved from the NCBI human genome GRCH37.p13; E-value <0.05) (Figure 3A), and we found 15 pairs of lncRNAs that are homologous between mice and humans (Table 2). To validate these findings, we analyzed the expression of these pairs in kidney tissues using real-time PCR; we found that the expression of the mouse lncRNA AU015836 was higher in the CaOx-exposed group than that in the control mice. Interestingly, the human homolog of AU015836, CHCHD4P4, was also found to be upregulated *in vitro* in the human proximal tubular epithelial cells that were exposed to COM compared to that in the untreated cells (Figure 3B). CHCHD4P4 is a human lncRNA that is 425 bp in length and is located on chromosome 3. Approximately 70% of the CHCHD4P4 lncRNAs were found in the nuclei, and the remainder were located in the cytoplasm (Figure 3C).

**Table 2.** Fifteen pairs of homologous long non-coding RNAs (lncRNAs).

Mouse/Human lncRNA	Mouse length	Human length	Total matching number	Mouse alignment		Human alignment		Style
				Initiation site	Termination site	Initiation site	Terminaton site	
n-Ts18-TRNAS23	82	82	82	1	82	1	82	Up
Gm5292-RPL15P3	669	702	655	23	524	20	524	Up
Gm7293-GAPDHP65	1259	1276	1008	53	1059	80	1078	Up
Gm5256-SLC25A5P5	1392	1101	906	263	1157	77	981	Up
Gm11223-STMN1P1	970	440	440	99	538	8	440	Up
5730408K05Rik-SNORA57	384	149	147	86	231	1	147	Down
1700024F13Rik-LOC101927497	669	466	326	335	659	136	461	Up
B230369F24Rik-RPL27AP6	2140	506	444	1	421	22	462	Down
Hoxa11as-HOXA11-AS	1839	1549	833	1062	1545	758	1250	Up
1500002F19Rik-RPL37P2	715	366	225	388	611	48	271	Down
Cdk3-ps-TEN1-CDK3	1427	3330	861	1	861	1836	2696	Up
AU015836-CHCHD4P4	3000	425	278	118	395	46	322	Up
A930017M01Rik-LOC644335	3350	4324	1193	370	875	370	871	Down
LOC102632352-LOC101928455	628	361	132	267	398	4	135	Down

### CHCHD4P4 promoted EMT in HK-2 cells that were exposed to COM

**Figure 4.**
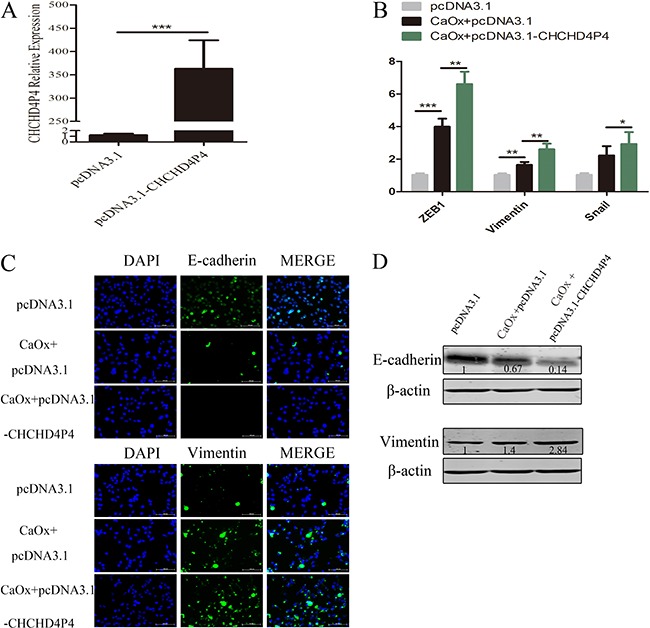
CHCHD4P4 overexpression. HK-2 cells were incubated with pcDNA3.1 encoding CHCHD4P4 cDNA (*A*) for 6 h for a stable transfection. HK-2 cells were treated with pcDNA3.1 or pcDNA3.1-CHCHD4P4 combined with calcium oxalate monohydrate (200 µg/mL) for 24 h. mRNA levels of the epithelial-mesenchymal transition (EMT) markers in the stable HK-2 cell clones (*B*). Protein levels (*D*) and immunofluorescence microscopic analysis (*C*) of the EMT markers in the indicated HK-2 cell clones. Scale bars = 100 µm. Data are reported a means±SD. *P<0.05, **P<0.01, ***P<0.001 (Student's *t*-test).

**Figure 5.**
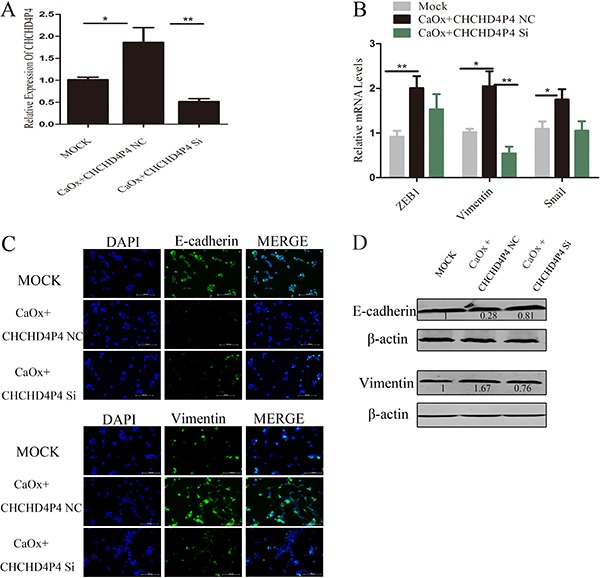
CHCHD4P4 silencing. HK-2 cells were transfected with the transfection agent (*A*) without siRNA (mock) or with siRNA against CHCHD4P4. mRNA levels (*B*), protein levels (*D*), and the results of the immunofluorescence microscopic analysis (*C*) of the EMT markers in the indicated HK-2 cells that were transfected with the siRNA against CHCHD4P4. Scale bars = 100 µm. Data are reported a means±SD. *P<0.05, **P<0.01 (Student's *t*-test).

To assess whether CHCHD4P4 regulates EMT, we first examined the effect of CHCHD4P4 on cellular phenotypes. The overexpression of CHCHD4P4 (Figure 4A) induced mesenchymal-like morphological features in the HK-2 cells and increased the transcript levels of *ZEB1*, *Vimentin*, and *Snail* (Figure 4B). The immunofluorescence and western blotting indicated that the overexpression of CHCHD4P4 resulted in higher vimentin protein levels and lower E-cadherin protein levels compared to the levels in the controls (COM-pcDNA3.1) (Figure 4C and D). More importantly, the silencing of CHCHD4P4 (Figure 5A) produced the opposite results (Figure 5B-D).

### CHCHD4P4 inhibited proliferation in HK-2 cells that were exposed to COM

**Figure 6.**
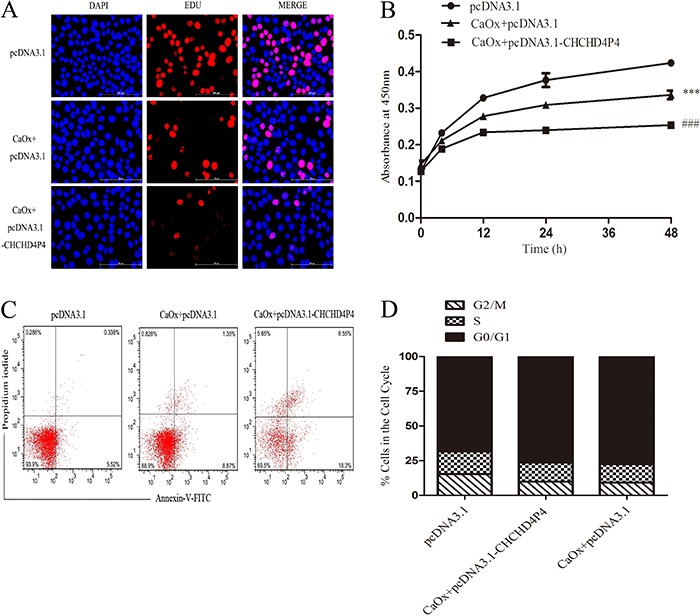
Effect of CHCHD4P4 on the cell cycle of HK-2 cells. Immunofluorescence microscopic analysis of 5-ethynyl-2'-deoxyuridine (EDU) (*A*) in the indicated HK-2 cell clones. Scale bars = 100 µm. Blue represents the nucleus, and red indicates the proliferation of cells. The CCK8 assay (*B*) was used to assess HK-2 cell proliferation (***P<0.001 *vs* pcDNA3.1, ^# ##^P<0.001 *vs* CaOx+pcDNA3.1, *t*-test). Annexin V flow-cytometric analysis of the HK-2 cells that were transfected with the pcDNA3.1 encoding CHCHD4P4 cDNA for 6 h and treated with calcium oxalate monohydrate for 24 h (*C*). Cell cycle analysis of the HK-2 cell clones (*D*).

**Figure 7.**
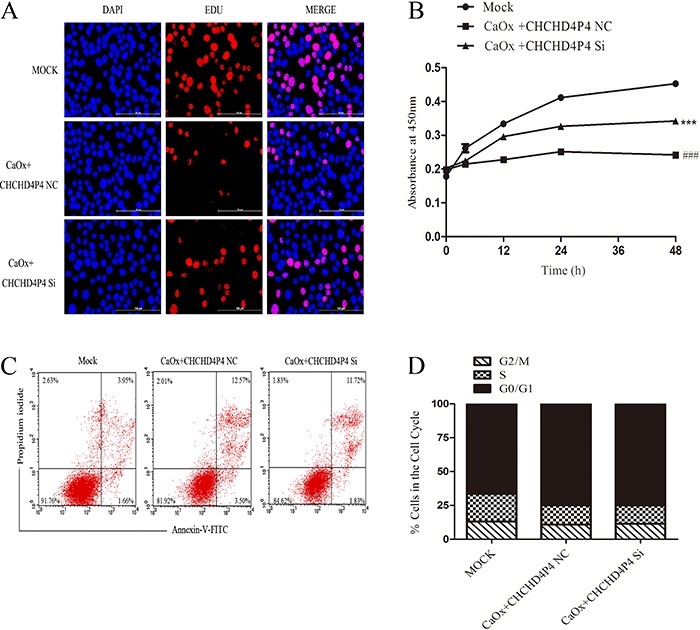
Effect of CHCHD4P4 on the cell cycle of HK-2 cells. Immunofluorescence microscopic analysis of 5-ethynyl-2'-deoxyuridine (EDU) (*A*) in the indicated HK-2 cell clones. Scale bars = 100 µm. Blue represents the nucleus, and red indicates the proliferation of cells. The CCK8 assay (*B*) was used to assess HK-2 cell proliferation (***P<0.001 *vs* mock treatment, ^# ##^P<0.001 *vs* COM+CHCHD4P4 NC, *t*-test). Annexin V flow-cytometric analysis (*C*), and cell cycle progression (*D*) of the HK-2 cells that were transfected with the siRNA against CHCHD4P4.

Immunofluorescence staining (Figure 6A) and the results of the CCK8 assay (Figure 6B) revealed that the CHCHD4P4 overexpression inhibited the proliferation of the HK-2 cells that were treated with COM. The inhibition was the most obvious following the COM+pcDNA3.1-CHCHD4P4 treatment. In our attempt to further investigate this result, we found that there were more apoptotic cells in the COM+pcDNA3.1-CHCHD4P4 group (Figure 6C). In contrast, only a slight difference was observed in the cell cycle progression (Figure 6D) between the COM+pcDNA3.1-CHCHD4P4 and COM+pcDNA3.1 samples. This result suggested that CHCHD4P4 possibly limits the HK-2 cell proliferation by promoting apoptosis *in vitro* and not by altering the cell cycle. Consistent with this result, the depletion of CHCHD4P4 produced the opposite effects (Figure 7A-D).

## Discussion

Renal stone disease is largely secondary to intra- or extra-renal urinary outflow obstruction, but crystal nephropathies can lead to significant kidney damage and renal failure (2,11). A decline in renal function is frequently accompanied by interstitial fibrosis, which is a common pathological occurrence in chronic kidney diseases (3,4). Therefore, the development of medical treatments that disrupt the progression of renal damage is important for protecting kidney function in patients with crystal nephropathies. Although the generation of experimental animal models of kidney stone disease has aided the study of these conditions (12–14), few effective therapeutic targets have been validated in clinical practice. An increasing number of studies have revealed that EMT is a key feature of kidney fibrosis (15–18). Injury to tubule epithelial cells (TECs) leads to a loss of functional parenchyma and induces evasive survival mechanisms, such as EMT initiation mediated through the TGF-β1-induced expression of Twist1 and Snail (19).

lncRNAs have been regarded as key regulators of genes at the epigenetic (20,21), transcriptional (22), and post-transcriptional (23) levels. To assess the effects of lncRNAs on gene expression, researchers have profiled genome-wide changes or changes in the expression level of individual genes after the knockdown or overexpression of a certain lncRNA. We hypothesized that the EMT-related genes are regulated by a specific lncRNA during renal damage caused by CaOx.

Through a microarray analysis, hundreds of lncRNAs were found to be differentially expressed (fold-change >1.5 and P<0.05) in the kidney tissues from mice that were exposed to glyoxylate compared with those from mice in the control group. The differentially expressed genes were classified into different functional categories according to the GO database for cellular components. The GO categories for the differentially expressed genes included extracellular region, proteinaceous ECM, ECM, extracellular space, basement membrane, and collagen. The pathway analysis results showed that only a subset of the differentially expressed genes was related to signaling pathways. These down- and upregulated genes were involved in cytokine-cytokine receptor interactions, ECM-receptor interactions, complement and coagulation cascades, and focal adhesion. The results of the GO and KEGG pathway analyses, therefore, indicated that the differentially expressed genes were related to ECM. This result is consistent with the hypothesis that the EMT comprises a critical series of events in which ECM and cell-cell interactions are altered to release epithelial cells from the surrounding tissue (24).

The genome sequences of mice and humans are highly divergent. Therefore, we applied the BLAST algorithm to mouse and human lncRNAs to identify homologous lncRNAs. Interestingly, we found that CHCHD4P4 is the human homolog of AU015836, and both genes were upregulated in the CaOx models compared to their levels in the respective control groups. We constructed stable CHCHD4P4-overexpressing and CHCHD4P4-knockdown HK-2 cells to evaluate the biological role of this molecule. We found that the majority of the CHCHD4P4 transcripts were distributed in the nuclei of the cells. Moreover, the mesenchymal phenotype was reduced, and expression of epithelial markers was promoted in the CHCHD4P4-knockdown HK-2 cells. In contrast, the exogenous expression of CHCHD4P4 promoted EMT. The EMT process is associated with a decrease in cell proliferation in various disease conditions, such as lung cancer (25), urinary obstruction (7), and colorectal cancer (26). Therefore, we sought to determine the functional relevance of the association between CHCHD4P4 and cell proliferation. Interestingly, we observed that CHCHD4P4 inhibited cell proliferation. Some studies have proven that apoptosis and EMT in TECs result in a cycle of damage and host response, leading to chronic fibrosis. Preventing EMT in injured TECs results in the preservation of their function, decreased pathological secretion, and alleviation of cell cycle arrest, all of which improve organ function (27,28). In our study, TECs exposed to COM showed EMT, which promoted apoptosis. However, the process was not associated with the cell cycle.

Furthermore, consistent with the result of a previous study (5), EMT occurred in the mice exposed to glyoxylate, which was not the case in the control mice. Our current work shows that the CHCHD4P4 lncRNA, which is the human homolog of AU01856, participated in the regulation of EMT, cell proliferation and apoptosis in COM-exposed HK-2 cells.

Our findings showed that CHCHD4P4 promoted EMT and inhibited cell proliferation in COM-exposed HK-2 cells, suggesting that CHCHD4P4 played a critical role in renal damage and nephrogenesis. Understanding the precise molecular mechanisms by which this lncRNA functions will be critical for exploring potential new strategies for the early diagnosis and treatment of kidney stone disease. Further research is necessary to explore the mechanism by which the EMT-related genes are regulated by CHCHD4P4.
